# Phase stability and microstructural properties of high entropy alloy reinforced aluminium matrix composites consolidated via spark plasma sintering

**DOI:** 10.1016/j.heliyon.2024.e24498

**Published:** 2024-01-14

**Authors:** Smith Salifu, Peter Apata Olubambi, Linda Teffo

**Affiliations:** a Centre for Nanoengineering and Advanced Materials, University of Johannesburg, South Africa; b Institute for Nanoengineering Research, Department of Chemical, Metallurgical and Materials Engineering, Tshwane University of Technology, Pretoria, South Africa

**Keywords:** High entropy alloy, Spark plasma sintering, Reinforcement, Phase stability, Microhardness

## Abstract

Spark plasma sintering (SPS) technique was employed in the consolidation of Cr_20_Mn_20_Ni_20_Cu_20_Nb_10_Co_10_ high entropy alloy (HEA) reinforced aluminium matrix composites. Phase stability and prediction expressions were used in the determination of the powder combination for the HEA. The microstructural analysis showed that an interdiffusion layer was formed between the aluminium matrix and the HEA particles in the sintered composites. Further investigation of the composites by X-ray diffraction (XRD) showed that in addition to the Al matrix phase present, other new phases (BCC, FCC and other intermetallics) were formed as a result of the reaction between the Al matrix and the atoms precipitated from the added HEA during sintering. The density of the HEA-reinforced Al matrix composites decreases with an increase in the wt.% of HEA from 98.6 % for pure aluminium to 98.1 % for the reinforced alloy with 10 % HEA, while the microhardness increases with an increase in the wt.% of the HEA from 35 HV for pure aluminium to 96.0 HV for the alloy reinforced with 10 % HEA.

## 1 Introduction

Over the years, metal matrix composites have gained significant interest in the world of research, and in the industries concerned with the development of metals and alloys [1]. This is due to the unprecedented material properties (unattainable via traditional alloys) achieved when metals are combined with other heterogeneous materials. The combination often results in improved strength, higher operating temperature [1,2] and invariably, improved lifespan. Aluminium-based composites have attracted great interest in the field of materials science and engineering and this is because they display exceptional properties such as high specific strength, lightweight, excellent resistance to corrosion and remarkable mechanical properties [3,4]. Based on these properties, aluminium-based composites have found extensive application in the automotive industry, defence and aerospace [5]. In the fabrication of aluminium-based composites, different kinds of materials such as metallic glass particles [6], ceramics [5], metallic compounds [7] and oxides [8] have been used as reinforcement.

The unique, and complex microstructure of HEAs set them apart from conventional alloys [9,10]. This unique microstructure displayed by HEAs is a key fact that determines their exceptional physical and mechanical properties. Depending on the compositions used, HEAs may exhibit a single-phase structure whereby all the elements that form the HEA are evenly distributed throughout the alloy or a multiphase structure that has distinct regions with different phases [9]. The phase stability of HEAs influences their microstructure such that some HEAs maintain their microstructure at high temperatures, thus, making them suitable for high-temperature applications, while some are not stable at high temperatures [10]. The ability to understand and control the microstructure of HEAs is very important for tailoring their properties to specific applications [11].

Unlike conventional alloys whose fabrications are single host metal-based, high entropy alloys (HEAs) are made up of several principal elements (usually, 5 and above elements) that show an exceptional combination of strength to ductility behaviour, good corrosion resistance, high fracture toughness [[12], [13], [14], [15], [16]], etc. Due to the excellent properties exhibited by HEAs, their use as reinforcement tends to broaden the particle-reinforcement metal composites range [[17], [18], [19], [20], [21]].

In a study conducted on copper-based composites using AlCoNiCrFe HEA particles as the reinforcement, it was discovered that 20 wt% AlCoNiCrFe HEA addition resulted in about 220 % increase in the compressive strength of the reinforced composite as compared to the copper matrix [21]. In another study, spark plasma sintering (SPS) technique was employed in the consolidation of Al0.6CoCrFeNi HEA particle-reinforced aluminium-based composites, and the fracture strength of the fabricated composites was high, in the range of 3120 ± 80 MPa [22]. Similarly, a study that compares the tensile strength, plasticity and elastic modulus of SiC–Al composite and CoNiFeAl0.4Ti0.6Cr0.5 HEA-reinforced Al-matrix composite showed that the HEA-reinforced Al-matrix composite gave a higher tensile strength, higher modulus of elasticity and better plasticity with the values 712 MPa, 171 GPa and 0.82 %, respectively [18].

Previous studies showed that Al-based composites consolidated or fabricated via the spark plasma sintering route display improved mechanical properties with excellent corrosion resistance [23]. Furthermore, CuZrAlTiNi HEA produced via mechanical alloying, followed by consolidation via SPS exhibited good pitting corrosion resistance in seawater and high microhardness with a value greater than 1000 HV [24]. The improvement in the properties exhibited by alloys and high entropy alloys reinforced metal composites fabricated via this route makes the technique popular in powder metallurgy.

Till date, the criteria for the formation of HEAs is still a subject of debate in the research world and different factors have been proposed to significantly influence the formation of these alloys [25]. According to Guo et al. [26], the combined effect of atomic size difference, mixing enthalpy, $\Delta H_{mix}$ and entropy, $\Delta S_{mix}$ determine solid solution formation in HEAs. In a study conducted by Chanda et al. [27], the formation of a two-phase solid solution is promoted by higher atomic radii difference in eutectic HEAs as the higher atomic radii difference increases the lattice stress that aids lattice distortion. In an attempt to distinguish intermetallic compound, solid solution and metallic glass, an atomic size-based mathematical model that accounts for the atomic packing misfitting in HEAs/multicomponent systems was reported Zhijun et al. [28]. At high temperatures, a significantly high change in entropy favours single-phase solid solution formation while an enthalpy with a high negative value favours the formation of dual-phase or two-phase solid solution that aids the stability of eutectic HEAs [27].

The relationship between dimensionless parameter (Ω), enthalpy ($\Delta H_{mix}$), and entropy ($\Delta S_{mix}$) was proposed by Yang et al. [29]; and by computing the relationship between Ω and atomic mismatch (δ), the criterion required for the prediction of stable solid state phase formation in HEAs was proposed. In HEAs, random single phase solid solution formation is predicted to be higher when high values of Ω are obtained while the opposite occurs when the value of Ω is low [9]. In the determination of the phases formed in HEAs, valence electron concentration (VEC) plays a crucial role [25,28], and the size or value of the VEC obtained during HEA design and development determines the different phases that would be formed [27].

In this study, phase stability and prediction expressions were used in the selection of elemental powders and design of Cr_20_Mn_20_Ni_20_Cu_20_Nb_10_Co_10_ HEA, and the SPS technique was employed in the consolidation of the HEA-reinforced aluminium matrix composites, thereby creating an opportunity to advance the fabrication of lightweight HEAs-reinforced metal matrix composite with improved properties.

## 2 Materials and methods

### 2.1 Materials

Commercial aluminium powder with 99.8 % purity was used as the matrix while chromium (99.2 %), manganese (99.6 %), copper (>99.0 %), niobium (99.8 %) and cobalt (99.5 %) powder particles with a size in the range ≤25μm were used in the fabrication of the HEA powder used as reinforcement in this study. The HEA chemical composition used in the study is depicted in Table 1, while the wt.% of the HEA and aluminium matrix used in the fabrication of the HEA-reinforced aluminium matrix composite is depicted in Table 2.

**Table 1:** Composition of HEA powder.

Elements	Cr	Mn	Ni	Cu	Nb	Co
Atomic %	20	20	20	20	10	10

**Table 2:** Weight % of aluminium and HEA used in the fabrication of the composites.

S/N	wt.% Aluminium	wt.% HEA
1	95.0	5.0
2	93.0	7.0
3	90.0	10.0

### 2.2 Experimental methods

**Fig. 1:**
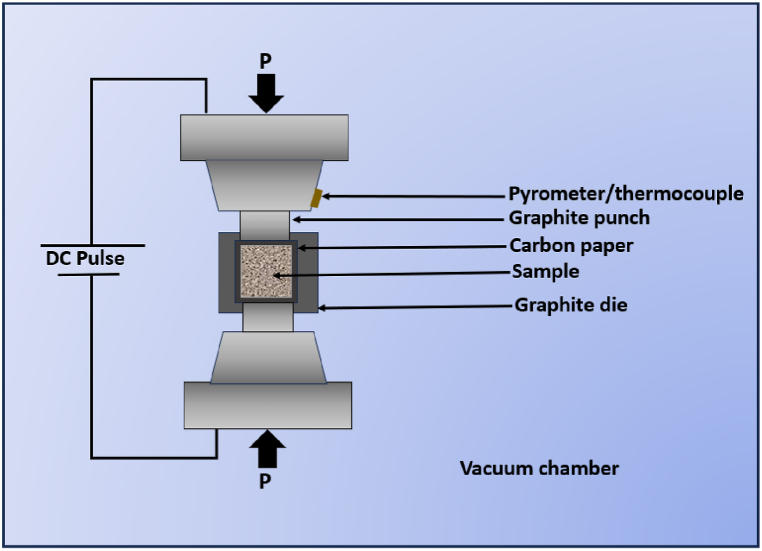
Schematic for the SPS technique used in the fabrication of the HEA-reinforced Al-matrix composites.

The elemental powders that make up the HEA were weighed as shown in Table 1 and dry milled in a planetary milling machine (Retsch Planetary Ball Mill PM 400) for 4 h at a milling speed of 200 RPM, 10 min break time at 30-min break intervals, at 10:1 ball-to-powder ratio. Thereafter, the milled HEA was mixed with aluminium powder in the ratio depicted in Tables 2 and in a modified tabular mixer at a 10:1 ball-to-powder ratio for 24 h, at a frequency of 50 Hz. The option of modified mixing of the HEA-reinforced Al matrix composite powder was opted for instead of dry milling because of the possible occurrence of cold welding during milling in the absence of a dry process control agent (PCA). SPS technique was employed in the consolidation of the powder particles of HEA-reinforced Al matrix composites. A 20 mm internal diameter high-strength and high heat-resistant die was used for the fabrication of the composite. The spark plasma sintering was done at a sintering temperature of 500°C, 100°C/min heating rate, and 10 min holding time, at a sintering pressure of 50 MPa. Fig. 1 depicts the fabrication schematic of the SPS process used in the fabrication of the composites.

The Vickers hardness of the sintered HEA-reinforced Al matrix composites samples was determined using a Microhardness tester with a load of 100 gf and a duration of 15 s. The hardness test was conducted on the samples 10 times and the average values of the indentation were obtained. In the determination of the density of the sintered HEA-reinforced Al matrix composite samples, the Archimedes method was used by an electronic analytical balance.

Furthermore, a PW1710 Philips, PANalytical Empyrean model X-ray diffractometer with Cu Ka radiation (λ = 0.15406 nm), accelerating current and voltage of 30.0 mA and 40.0 kV, respectively and scanning speed of 1.50 deg/min and scanning range between 5° and 90° was employed for the phase analysis of the powders and sintered composites. An energy-dispersive equipped Scanning Electron Microscope (FE-SEM: JEOL JSM 7600F) was employed for the microstructural characterization of the fabricated samples.

### 2.3 Spark plasma sintering

**Fig. 2:**
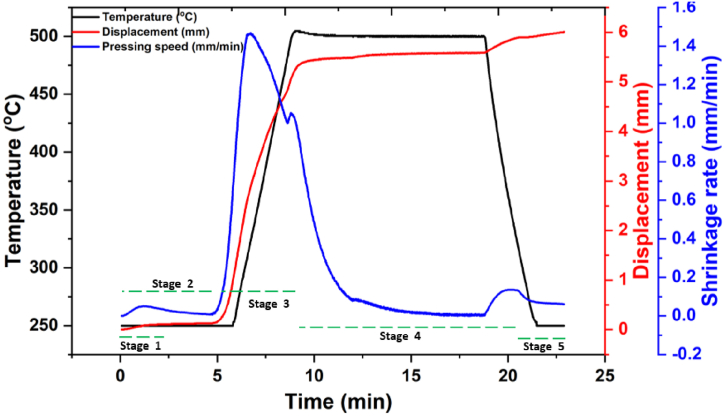
Sintering profile for the consolidation of HEA-reinforced Al matrix composites.

According to the recorded spark plasma sintering data for the HEA-reinforced Al matrix composites depicted in Fig. 2, there is a relationship between the sintering time and temperature; sintering time and the speed/displacement of the punch. From the figure, it was observed that both the displacement and temperature curve start from 0 min, however, the sintering temperature starts at 250°C, being the initial temperature of the furnace as read by the sensitive pyrometer monitoring the temperature (sintering) within the furnace. In Fig. 2, five different stages are observed, with the first stage occurring between 0 and 2 min. This stage was initiated by spark creation between the particles and the gas removal from the particles of the powders being sintered, without significant movement of the punch [30]. In the second stage, rearrangements and localized deformation of powder particles occur, with a linear displacement of the punch as a function of the increase in temperature up to 255°C, and lasted for about 4 min (1–5 min). Powder particle compaction, necking formation and bulk deformation of the powder particles are the characteristics of the third stage, and this stage occurs for about 3 min (5–8 min). A rapid movement in the punch was observed as the sintering temperature of the powder increased from 255°C to 475°C. However, at 475°C, the displacement of the punch remained constant as the applied pressure/pressing speed did not lead to further particle deformation. This stage lasted for about 10 min, and neck growth via the phenomenon of mass transport occurred [31,32]. Thus, the sintering of the powder particles finally occurs as the voids between adjacent necks are closed by the increasing movement of the punch, and the shrinking of the sintered sample due to the applied pressure.

## 3 Results and discussion

### 3.1 Stability and phase prediction

Guo et al. [33,34] and Yang [35] predicted respectively, the mechanism for the formation of BCC/FCC phase stability and solid solution in HEAs. According to the authors, the competition between enthalpy and entropy can be determined through the use of a unitless entity with the symbol, Ω as depicted in Equation (1). This expression was employed to determine/predict the solid solution formation in the HEA developed.

(1) $$\Omega =\frac{T_{m}\Delta S_{mix}}{\Delta H_{mix}}$$

where the terms $T_{m}$ represents the melting temperature, $\Delta S_{mix}$ represents the Mixing entropy and $\Delta H_{mix}$ represents the mixing enthalpy.

For solid solutions, the Ω value needs to be greater than or equal to 1.10 (Ω≥1.10). Also, the atomic mismatch (δ) between the elements that make up the HEA can be computed using the expression:

(2) $$\delta =\sqrt{\sum_{i=1}^{n}{\left(c_{i}\left(1-r_{i}/\bar{r}\right)\right)}^{2}}$$

where $c_{i}$ represents the ith element atomic ratio, $r_{i}$ represents the ith element radius and $\bar{r}$ represents the average atomic radius.

To promote solid solution formation in HEAs, the atomic radii of the elements making up the HEA need to be similar or close, and the value of δ must be less than or equal to 6.6 % (δ≤6.6%) [33].

The mixing enthalpy of the HEA can be computed using the expression:

(3) $$\Delta H_{mix}=\sum_{i=1,i\neq j}^{n}4\Delta H_{ij}^{mix}c_{i}c_{j}$$

where $\Delta H_{ij}^{mix}$ represents the ith and jth element mixing enthalpy.

Using the expression in Equation (4) the melting temperature of the HEA can be computed:

(4) $$T_{mix}=\sum_{i=1}^{n}c_{i}{\left(T_{m}\right)}_{i}$$

where ${\left(T_{m}\right)}_{i}$ represents the ith element melting point. Furthermore, the entropy of the HEA is calculated using:

(5) $${\Delta S}_{mix}=-R\sum_{i=1}^{n}\left(c_{i}\;\ln\;c_{i}\right)$$

where R represents the gas constant (8.314 J/K.mol).

From these equations, high entropy alloys are considered stable if the condition $-15<{\Delta H}_{mix}<5\;kJ/mol$, $12\leq \Delta S_{mix}\leq 17.5\;J/K$ alongside other important conditions initially stated are met [29,36].

Based on the enthalpy pair of the elements that make up the HEAs [37] and the expression for the mixing enthalpy (Equation (3)), the mixing enthalpy for the HEA used in reinforcing the aluminium matrix in this study was computed as shown in Table 3.

**Table 3:** Binary atomic pairs mixing enthalpy for the Cr_20_Mn_20_Ni_20_Cu_20_Nb_10_Co_10_ HEA.

Elements	Enthalpy of mixing (kJ/mol)					
	Cr (0.2)	Mn (0.2)	Ni (0.2)	Cu (0.2)	Nb (0.1)	Co (0.1)
**Cr (0.2)**	0					
**Mn (0.2)**	2	0				
**Ni (0.2)**	−7	−8	0			
**Cu (0.2)**	12	4	4	0		
**Nb (0.1)**	−7	−4	−30	3	0	
**Co (0.1)**	−4	−5	0	6	−26	0
			**Total Enthalpy of mixture** = **-3.16**			

The stability of the phases present in HEAs was predicted by adopting the valence electron concentration (VEC) expression given as:

(6) $$VEC=\sum_{i=1}^{n}c_{i}{\left(VEC\right)}_{i}$$

According to the expression, VEC≥8 indicates a stable FCC phase, VEC≤6.87 indicates a stable BCC phase while both FCC and BCC phase is found when 6.87<VEC<8 [[37], [38], [39]].

Adopting the expression for VEC in Equation (6) and the other expressions (Equations (1), (2), (4), (5)), the phase prediction and stability of the HEA used in this study were determined, and the summary of the obtained values are depicted in Table 4.

**Table 4:** Summary of the phase prediction and stability of the developed HEA.

Parameters	Predicted value
Enthalpy of mixture, ${\Delta H}_{mix}$	3.16 kJ/mol
Melting temperature of mixture, $T_{mix}$	1535 °C
Entropy of mixture, ${\Delta S}_{mix}$	14.21 J/K
Omega, Ω	6.90
Deta, δ	5.53 %
Valence Electron Concentration, VEC	8.2

### 3.2 Microstructure

**Fig. 3:**
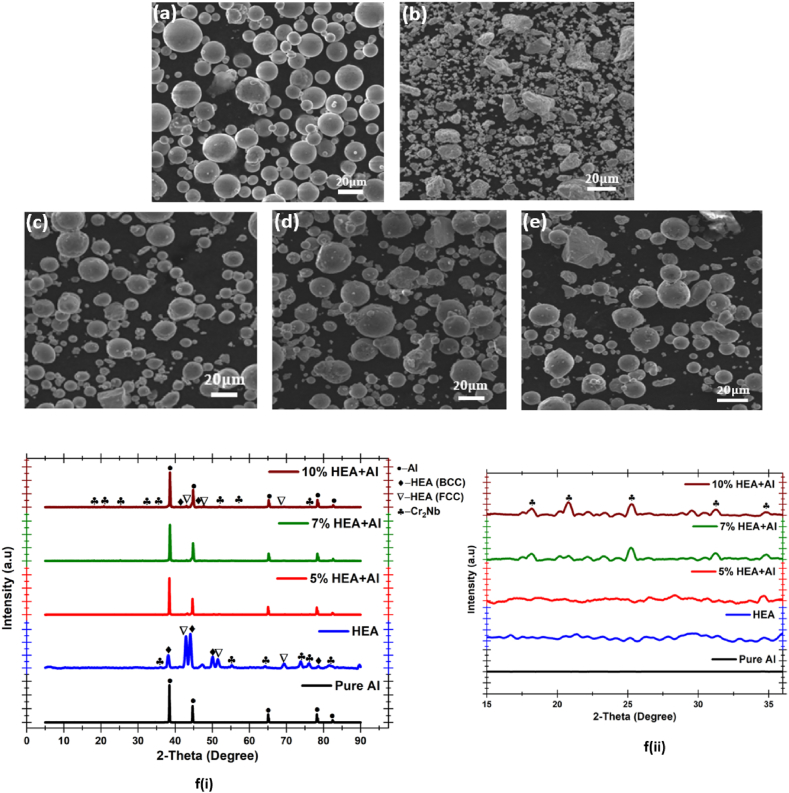
SEM image of (a) aluminium powder, (b) HEA powder, (c) 5 % HEA + Al powder, (d) 7 % HEA + Al powder, (e) 10 % HEA + Al powder, and (f) XRD of (i) the corresponding powders and (ii) magnification of 2-theta around 15–35°.

Depicted in Fig. 3 are the SEM micrograph and the XRD pattern of the milled HEA powder particles. The SEM of the aluminium particles in Fig. 3(a) shows that the powder consists of smooth-surface spherical microparticles that are well-dispersed. A similar aluminium powder micrograph with well-dispersed microparticles was reported by Chen et al. [40]. From the SEM micrograph of the milled HEA shown in Fig. 3(b), it was observed that the powders are relatively uniform, though with a noticeable mixture of spherical, flat and potato-like shapes. A similar shape mixture or irregularity in HEA powder was reported by Yim et al. [41], and these irregularities or noticeable shape alterations in the morphology of the milled powder are capable of significantly influencing the properties (physical and mechanical) of the HEA and the HEA-reinforced Al composite matrix [42] since the irregularity is an indication that they have appreciable powder flowability [43,44]. Fig. 3(c–e) show the micrograph of the powders of the HEA-reinforced Al matrix composites as a function of an increase in the wt.% of HEA. In the three composite powders, satellite spheres are noticed on the surface of the powder particles having larger particle sizes. It was also observed that the larger the particle size, the rougher the surface of the powder. The XRD pattern of the Al, HEA and HEA-reinforced Al matrix composite is depicted in Fig. 3 (f). The diffraction peaks showed that all the elements that make up the HEA and HEA-reinforced Al matrix composites are present. Furthermore, the milling/mixing time and speed used for the preparation of the HEA and HEA-reinforced Al matrix composite resulted in the formation of BCC and FCC structure HEA, and Cr_2_Nb intermetallic phase, as indicated by the XRD peaks. A similar XRD powder pattern was reported by Liu et al. [45] and Yuan et al. [46] when they ball-milled CoCrCuFeNi high-entropy alloy from 30 min to 6 h, and when CoCrFeNiMo_0.2_ reinforced titanium matrix composites powder were ball-milled, respectively.

**Fig. 4:**
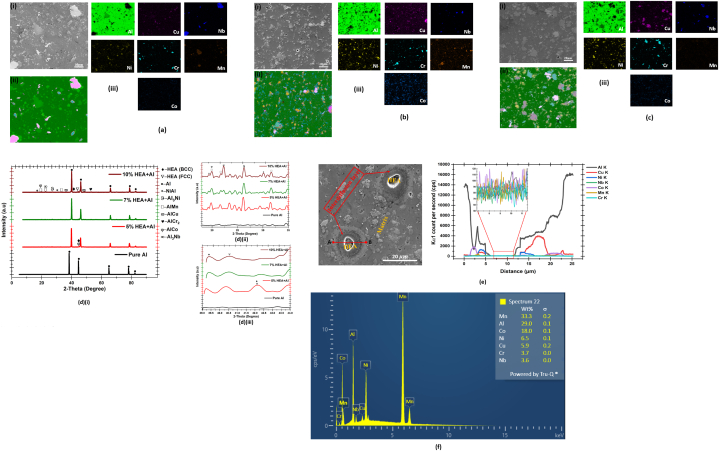
(a) (i) SEM image (ii), EDS phase mapping (iii) EDS elemental mapping of 5 % HEA reinforced Al matrix composites; (b) (i) SEM image (ii), EDS phase mapping (iii) EDS elemental mapping of 7 % HEA reinforced Al matrix composites; (c) (i) SEM image (ii), EDS phase mapping (iii) EDS elemental mapping of 10 % HEA reinforced Al matrix composites; (d) XRD of (i) the sintered samples (ii) magnification of 2-theta around 18–35°, and (iii) magnification of 2-theta around 39–44°; (e) line scan EDS and plot showing a region of interdiffusion layers in sintered 5 % HEA reinforced Al matrix composite; and (f) point EDS in area A, showing elemental distribution in 5 % HEA-reinforced Al matrix composite.

The SEM micrograph, elemental mapping and XRD of the sintered Cr_20_Mn_20_Ni_20_Cu_20_Nb_10_Co_10_ HEA-reinforced Al matrix composites are depicted in Fig. 4. Based on the elemental mappings, the dispersion state of the HEA Cr, Mn, Ni, Nb and Co is uniform. Thus, the Cr_20_Mn_20_Ni_20_Cu_20_Nb_10_Co_10_ HEA distributes evenly in the Al matrix during mixing and this greatly influences the quality of the samples sintered. In addition, the average size of the HEA particles is close to that of Al, thus, contributing to the improvement in the densification of the sintered samples [1]. As depicted in Fig. 4a(i), 4b(i) and 4c(i), the microstructure of the HEA-reinforced Al matrix composites consists of a continuous grey matrix phase and white particle phase. The HEA particles consisting of different sizes are distributed evenly in the Al matrix in order to form a typical composite structure. Since the particles of the HEA did not undergo severe deformation during mixing and milling, there was diffusion between the particles of the HEA and the matrix as indicated by the obvious diffusion layer seen between the matrix and HEA particles in the composites (as shown by the EDS line scan and plot in Fig. 4(e)), and the diffusion layer exhibited two different colours with the outer diffusion layer closer to the HEA having a dark grey-like colour while the inner diffusion layers have a light grey-like colour. The magnitude of the k factor determines the content of the elements at every region along the scan and the intensity of each element is determined by the fluctuation of the k factor. At the zoomed region (between 7 and 12 μm) on the line scan plot in Fig. 4(e), It was observed that the distribution of all the elements that make up the HEA-reinforced Al matrix composites are approximately the same. A similar diffusion layer was reported by Zhanwei et al. [46] when CoCrFeMnNi HEA particles were used to reinforce 2024Al. Relatively, the diffusion layer on the side in contact with HEA is flat while on the side in contact with the Al matrix, the diffusion layer is serrated. This behaviour could be attributed to the uneven diffusion of the elements during sintering as atoms are known to randomly jump in all directions during diffusion. This leads to a concentration gradient between the same elements at different interfacial locations that result in solid-liquid interfacial interaction not occurring simultaneously during sintering [46]. Thus, the region with high element concentration on the interface reacts first and grows quickly. The point EDS elemental spectrum (Fig. 4(f)) of the sintered 5 % HEA-reinforced Al matrix composite at point A (Fig. 4(e)) confirms the presence of all the elements that make up the composites, with point A being Mn-rich region.

The XRD pattern for the spark plasma sintered Al and HEA-reinforced Al matrix composites is depicted in Fig. 4(d). A slight detectable shift was observed in the XRD peak of the HEA-reinforced Al matrix. This shift is due to the induced stress and strain in the crystal lattice caused by the introduction of the HEA. The induced stress affects the interplanar spacing and, consequently, the XRD pattern. For the sintered aluminium, all the picks are aluminium. Thus, signifying the absence of noticeable contamination during the sintering process. During the sintering of the HEA-reinforced Al matrix composite powder, the atoms (Cu, Co, Cr, Nb, Mn, and Ni) of the elements that make up the HEA and Cr_2_Nb formed during milling were precipitated during heating and sintering and these precipitated atoms react with the surrounding Al upon the activation of the discharged plasma to form new phases [46], as depicted in the XRD result (Fig. 4(d)). The formation of the new phases is an indication that there is a certain degree of interfacial interaction between the HEA reinforcement and the Al matrix during sintering. For the composites, there was the formation of new phases in addition to the aluminium matrix during the course of sintering due to the addition of the HEA. In all the sintered composites, a small amount of noticeable BCC and FCC HEA phases were seen, and new phases such as NiAl, Al_3_Ni, AlMn, AlCu, AlCr_2_, AlCo and Al_3_Nb were formed in the bulked sintered composites.

### 3.3 Density and microhardness

**Fig. 5:**
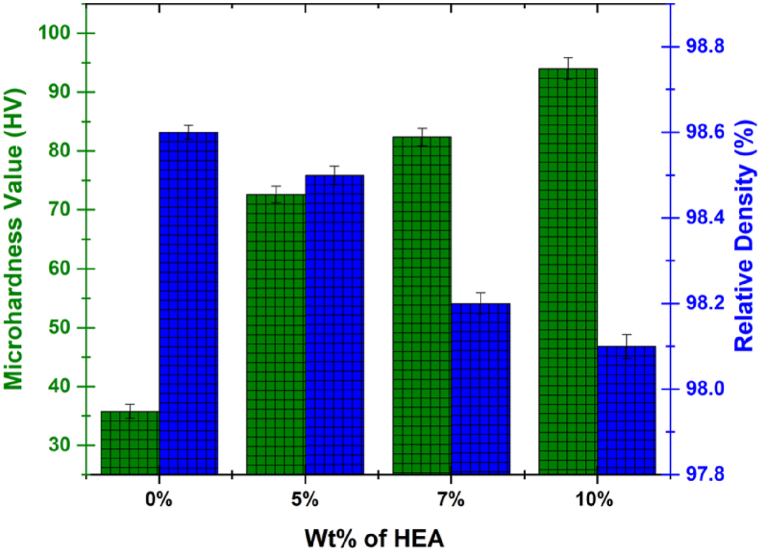
Effects of HEA on the density and microhardness of HEA-reinforced Al matrix composites.

The relative density and microhardness of the sinters HEA reinforced Al-matrix composites as a function of the increase in the wt.% HEA content is depicted in Fig. 5. From the figure, it was observed that the microhardness of the HEA-reinforced Al matrix composites increases with an increase in the wt.% of HEA while the value of the relative density was observed to decrease with an increase in the wt.% of the HEA.

The microhardness value of the sintered pure aluminium is 35.8 HV. However, the addition of as low as 5 wt% HEA to Al resulted in about 202.8 % increase in the microhardness value (72.6 HV). At 7 wt% and 10 wt% addition of HEA to Al, the obtained microhardness values obtained are 82.4 and 96.0 HV, which corresponds to about 230 % and 268 % increase in the microhardness, respectively, when compared with the pure aluminium. The significant increment observed in the microhardness values when HEA was introduced or added to the pure aluminium to form the HEA-reinforced Al matrix composite is not only due to the unique properties of the HEA used as reinforcement but also due to the presence of interdiffusion layer which helps in the reduction of sintering defects and thus, further increase the hardness of the composites [46].

Although the adopted spark plasma sintering technique used in the consolidation of the HEA-reinforced Al matrix composites improves the densification of the composites, the densification of the composites was observed to slightly decrease with an increase in the addition of HEA as shown in Fig. 5. The reason for this behaviour could be attributed to the increase in inhomogeneity of the atoms with an increase in the quantity of HEA, as this inhomogeneity tends to affect the atomic packing, support the formation of pores and consequently decrease the densification of sintered samples [47]. For the spark plasma sintered samples containing 0, 5, 7 and 10 wt% HEA, 98.6, 98.5.98.2 and 98.1 % relative densities were achieved, respectively. This result shows that high densification was achieved courtesy of the fabrication technique used. However, the addition of HEA as a reinforcement did not improve the densification of the composites.

## 4 Conclusion

In this study, spark plasma sintering technique was employed in the consolidation of high entropy alloy-reinforced aluminium matrix composites. Phase prediction/stability expressions were employed in the determination of the phase stability of the Cr_20_Mn_20_Ni_20_Cu_20_Nb_10_Co_10_ HEA before using it as reinforcement in the matrix. The sintered composites consist of a matrix, HEA particles and an interdiffusion layer. During the sintering of the HEA-reinforced Al powders, atomic precipitation from the HEA solid solution occurs and these atoms react with the surrounding Al matrix to form new phases. The new phases formed are an indication of interfacial interaction that occurs during sintering. BCC and FCC phase HEA crystal structures and intermetallic were compounds formed upon the addition of HEA to the Al matrix. These new phases and intermetallics formed are due to the reaction between the matrix and the elements that formed the HEA. In all the HEA-reinforced Al matrix composites formed, a small amount of both BCC and FCC HEA phases were present. However, the presence of these phases was more pronounced in the composite with 10 % HEA. The relative density of the sintered composite decreases with an increase in the wt.% of HEA (ranging from 98.6 % for pure Al to 98.1 % for 10 % HEA-reinforced Al), thus, indicating that the addition of the HEA reduces the densification of the sintered composites. This behaviour is due to the high inhomogeneity in the atomic structure, which resulted in the poor atomic packing experienced by the elements during sintering. Nevertheless, the room temperature microhardness of the composites was significantly increased with an increase in the wt.% of HEA, such that the composite with 10 wt% HEA displayed the highest microhardness with a value of 96.0 HV while the pure aluminium had a microhardness value of 35.8 HV. This behaviour could be attributed to the presence of more BCC phases in the composite with 10 % HEA since BCC-dominated materials tend to display higher hardness than their FCC counterpart at room temperature.

## Data availability

Data will be made available upon request.

## Ethics approval

Not applicable.

## Consent to participate

Not applicable.

## Consent for publication

All authors have read and agreed to publish the manuscript. Competing interests The authors declare no competing interests.

## CRediT authorship contribution statement

**Smith Salifu:** Writing - original draft, Methodology, Investigation, Conceptualization. **Peter Apata Olubambi:** Writing - review & editing, Supervision. **Linda Teffo:** Writing - review & editing, Investigation.

## Declaration of competing interest

The authors declare that they have no known competing financial interests or personal relationships that could have appeared to influence the work reported in this paper.
